# Connective Tissue Growth Factor Is Overexpressed in Explant Lung Tissue and Broncho-Alveolar Lavage in Transplant-Related Pulmonary Fibrosis

**DOI:** 10.3389/fimmu.2021.661761

**Published:** 2021-05-25

**Authors:** Arno Vanstapel, Roel Goldschmeding, Roel Broekhuizen, Tri Nguyen, Annelore Sacreas, Janne Kaes, Tobias Heigl, Stijn E. Verleden, Alexandra De Zutter, Geert Verleden, Birgit Weynand, Erik Verbeken, Laurens J. Ceulemans, Dirk E. Van Raemdonck, Arne P. Neyrinck, Helene M. Schoemans, Bart M. Vanaudenaerde, Robin Vos

**Affiliations:** ^1^ Department of Chronic Diseases and Metabolism, Katholieke Universiteit, Leuven, Belgium; ^2^ Department of Pathology, University Hospital Leuven, Leuven, Belgium; ^3^ Department of Pathology, University Medical Center Utrecht, Utrecht, Netherlands; ^4^ Department of Microbiology, Immunology and Transplantation, Katholieke Universiteit, Leuven, Belgium; ^5^ Department of Respiratory Diseases, Lung Transplant Unit, University Hospital Leuven, Leuven, Belgium; ^6^ Department of Thoracic Surgery University Hospital Leuven, Leuven, Belgium; ^7^ Department of Cardiovascular Sciences, Katholieke Universiteit, Leuven, Belgium; ^8^ Department of Anesthesiology, University Hospital Leuven, Leuven, Belgium; ^9^ Department of Hematology, University Hospital Leuven, Leuven, Belgium

**Keywords:** GVHD, RAS, BOS, lung transplantation, fibrosis, CTGF, CLAD

## Abstract

**Background:**

Connective tissue growth factor (CTGF) is an important mediator in several fibrotic diseases, including lung fibrosis. We investigated CTGF-expression in chronic lung allograft dysfunction (CLAD) and pulmonary graft-versus-host disease (GVHD).

**Materials and Methods:**

CTGF expression was assessed by quantitative real-time polymerase chain reaction (qPCR) and immunohistochemistry in end-stage CLAD explant lung tissue (bronchiolitis obliterans syndrome (BOS), n=20; restrictive allograft syndrome (RAS), n=20), pulmonary GHVD (n=9). Unused donor lungs served as control group (n=20). Next, 60 matched lung transplant recipients (BOS, n=20; RAS, n=20; stable lung transplant recipients, n=20) were included for analysis of CTGF protein levels in plasma and broncho-alveolar lavage (BAL) fluid at 3 months post-transplant, 1 year post-transplant, at CLAD diagnosis or 2 years post-transplant in stable patients.

**Results:**

qPCR revealed an overall significant difference in the relative content of CTGF mRNA in BOS, RAS and pulmonary GVHD vs. controls (p=0.014). Immunohistochemistry showed a significant higher percentage and intensity of CTGF-positive respiratory epithelial cells in BOS, RAS and pulmonary GVHD patients vs. controls (p<0.0001). BAL CTGF protein levels were significantly higher at 3 months post-transplant in future RAS vs. stable or BOS (p=0.028). At CLAD diagnosis, BAL protein content was significantly increased in RAS patients vs. stable (p=0.0007) and BOS patients (p=0.042). CTGF plasma values were similar in BOS, RAS, and stable patients (p=0.74).

**Conclusions:**

Lung CTGF-expression is increased in end-stage CLAD and pulmonary GVHD; and higher CTGF-levels are present in BAL of RAS patients at CLAD diagnosis. Our results suggest a potential role for CTGF in CLAD, especially RAS, and pulmonary GVHD.

## Introduction

Connective tissue growth factor (CTGF, CCN2), a cysteine-rich matricellular protein, is an important profibrotic mediator in several fibrotic disorders, including idiopathic pulmonary fibrosis (IPF) (1, 2). In IPF, promising results of the PRAISE trial were recently published, which was a phase II randomized trial of Pamrevlumab (FG-3019), a fully recombinant human monoclonal antibody against CTGF, demonstrating attenuation of disease progression (2).

Currently, no data are available regarding the potential role of CTGF in the development of chronic lung allograft dysfunction (CLAD) after lung transplantation (LTx); nor in lung graft-versus-host disease (GVHD) after allogeneic hematopoietic stem cell transplantation (HCT). However, as in IPF, fibrotic lung remodeling forms the pathologic hallmark of both CLAD and pulmonary GVHD.

CLAD affects more than 50% of patients within 5 years after lung transplantation (LTx) (3, 4) and remains the major limitation to long term graft survival. CLAD is a clinical diagnosis, defined by a persistent decline in forced expiratory volume in one second (FEV1) of at least >20% compared to baseline. There are currently two main phenotypes described, which are both characterized by distinct fibrotic remodeling (5). Bronchiolitis obliterans syndrome (BOS) is characterized by an obstructive pulmonary function deficit and air trapping on computed tomography (CT) (5). The pathologic hallmark of BOS are bronchiolitis obliterans (BO) lesions, which are characterized by sub-epithelial fibrosis of the small airways. Although several risk factors have been identified for CLAD development (e.g. infection, cellular rejection), the underlying pathogenesis remains largely unknown, but likely results from a complex interplay and dysregulation of pro- and anti-fibrotic mediators. Molecular analysis revealed distinct gene expression patterns, with upregulated transforming growth factor beta (TGF-β), fibroblast growth factor-2, tumor necrosis factor-α and endothelin-1, which interfere with the mediators of myofibroblast homeostasis and extracellular matrix turnover [e.g. matrix metalloproteinases (MMPs) and tissue inhibitors of metalloproteinases (TIMPs)] (6–8).

The second main phenotype of CLAD, restrictive allograft syndrome (RAS), is defined by a restrictive pulmonary function deficit [i.e. >10% decline in total lung capacity (TLC))]and the presence of persistent radiological opacities (9, 10). Pleuroparenchymal fibro-elastosis forms the dominant fibrosis pattern in RAS and non-specific interstitial pneumonia, another fibrotic pattern, is observed in approximately a quarter of RAS patients (11). Interestingly, BO lesions are also present in the majority of end-stage RAS explant lungs, indicating potential overlap of BOS and RAS regarding the underlying fibrogenesis.

CLAD further shows considerable clinical, histopathologic and molecular overlap with pulmonary graft-versus-host-disease (GVHD) after HCT, including development of BO, pleuroparenchymal fibro-elastosis, and an adverse outcome (12, 13). Molecular analysis previously revealed large overlap in the fibrosis-associated gene expression signatures (e.g. MMP, TGF-β) of CLAD vs. pulmonary GVHD (14), indicating potential overlap in pathophysiology.

Since the development of fibrotic lung lesions is central in the pathogenesis of both CLAD and pulmonary GVHD, and given the afore mentioned advancements in CTGF as a promising therapeutic target in IPF, we tested the hypothesis that CTGF may play a role in CLAD and pulmonary GVHD. We therefore analyzed CTGF expression in end-stage lung tissue of well-defined BOS, RAS, pulmonary GVHD and control lungs. In addition, we explored the potential of CTGF as a biomarker, by analyzing CTGF protein expression in broncho-alveolar lavage (BAL) and plasma of CLAD and stable LTx patients.

## Materials and Methods

### CLAD and Pulmonary GVHD Diagnosis

CLAD was defined as per recent ISHLT consensus guidelines (5, 10). CLAD was defined by a persistent (> 3 months) decline in FEV_1_ of at least 20% compared to the best post-operative baseline (mean of the two highest FEV_1_ values post-LTx taken at least 3 weeks apart), in absence of another cause of pulmonary function decline (e.g. infection) (5). BOS was diagnosed when patients presented with a purely obstructive lung function deficit (i.e. ≥20% FEV_1_ decline with FEV_1_/FVC ratio of <0.7, and <10% TLC decline), and without persistent radiological opacities. RAS was diagnosed based on the presence of a restrictive pulmonary function decline (i.e. an additional decline in TLC of ≥10% compared to the best baseline post-LTx, or if unavailable, a decline in FVC of ≥20%) in combination with the presence of persistent opacities on chest CT (10). Stable LTx recipients were defined by a stable lung function (i.e. <10% FEV_1_ decline, <10% forced vital capacity (FVC) decline, and <10% TLC decline) for the entire available follow up period, with a minimum of at least three years post-LTx follow up available. Graft loss was defined as death or redo-transplantation (follow-up until July 2020). Pulmonary GVHD was diagnosed by an experienced hematologist, according to the 2014 NIH definition (15). Briefly, this included lung function testing, CT evaluation and clinical symptom assessment.

### Explant Lung Tissue Selection

Fresh-frozen explant lung tissue (obtained at redo-LTx or autopsy), collected as previously described and stored in our lung research biobank (16), was used for quantitative real-time polymerase chain reaction (qPCR) analysis of CTGF mRNA levels. We included CLAD patients with BOS (n=20) and RAS (n=20). As no explant tissue was available from stable LTx patients, unused donor lungs (n=20) were matched for age and gender and served as control group. In addition, fresh-frozen explant lung tissue was included from patients suffering from pulmonary GVHD after allogeneic HCT (n=9), which was obtained at LTx or autopsy. A flowchart of patient and tissue selection is provided in **Supplementary Figure S1**. For the immunohistochemical analysis, formalin-fixed and paraffin-embedded (FFPE) explant tissue samples, previously obtained for diagnostic purposes, were retrieved from the pathology archives. For 6/20 (30%) BOS lungs and 7/20 (35%) RAS lungs included in the qPCR analysis, no FFPE material was available in the pathology archives. Therefore, additional CLAD patients were included following the same criteria. In addition, FFPE explant material from all non-transplanted donor lungs (n=20) and pulmonary GVHD patients (n=9) included in the qPCR analysis was available and included in the immunohistochemical analysis.

### qPCR Analysis

RNA extraction of fresh-frozen lung tissue was performed by placing the tissue in Trizol reagent (Thermo Fisher), with subsequent disruption using inert zirconium beads and a tissue homogenizer (Precellys). RNA was isolated from the Trizol fractions and further purified using the RNeasy Mini Kit (Qiagen). cDNA synthesis from 3 µg RNA was done using Superscript III reverse transciptase (Thermo Fisher). qPCR was performed with a ViiA7 real-time PCR system (Thermo Fisher), using TaqMan Gene Expression Assays for CTGF (ThermoFisher Hs00170014_m1), TBP (ThermoFisher Hs99999905_m1, and GAPDH (ThermoFisher Hs00427620_m1). Amplification reactions were run in duplicate and analyzed using ViiA7 Software (version 1.2.4). CTGF expression was analyzed using the comparative cycle threshold method, and normalized to the geometric mean of the two housekeeping genes (TBP and GAPDH).

### CTGF Immunohistochemistry and Histological Analysis

A representative 3µm tissue section of FFPE material was used for CTGF immunohistochemistry (sc-14939; Santa Cruz Biotechnology Inc., Heidelberg, Germany), as previously described (17). Tissue slides were subsequently digitalized using a Nanozoom XR scanner (Hamamatsu Photonics, München, Germany) and assessed through digital pathology slide viewing software (Aperio ImageScope version 12.4.3., Leica Biosystems, Wetzlar, Germany). CTGF expression of the respiratory epithelium was assessed by a semi-automated approach using open-source image analysis QuPath software (18). The respiratory epithelium was manually delineated and subsequently automatically analyzed using the ‘*positive cell detection*’ tool, that uses an automated cell segmentation algorithm to identify (positive) cells. After visual confirmation and optimization of the different thresholds for cell detection and staining intensities (i.e. negative, 1+, 2+ and 3+), thresholds were consistently applied for all slides. In addition, CTGF expression patterns of other cell types (e.g. endothelial cells, fibroblasts) and further histological findings were reviewed qualitatively by an experienced pulmonary pathologist.

### LTx Patient Selection for BAL and Blood Analysis

Sixty patients who underwent LTx at the University Hospitals Leuven between 2007 and 2018 were included for BAL and plasma analysis in this study (**Supplementary Figure S1B**). Patient inclusion was based on retrospective diagnosis of CLAD following the above stated criteria. We selected BOS (n=20), RAS (n= 20), and stable LTx recipients (n=20). BOS and RAS patients were matched with stable LTx recipients for age, gender, and underlying native lung disease; and further selected based on the availability of BAL and plasma samples at the different matched time points. For 2/20 (10%) BOS patients and 8/20 (40%) RAS patients, fresh-frozen and FFPE explant lung tissue was also included.

### BAL Collection

All LTx recipients received routine follow-up visits at fixed time points, as described earlier (19). BAL samples, obtained at three different time points and stored at -80°C, were included in this study: at 3 months post-LTx, 1 year post-LTx, and at CLAD diagnosis for BOS and RAS patients, or at 2 years post-LTx for stable LTx recipients (i.e. the last routine bronchoscopic evaluation and the closest matched time point as comparison for BAL samples at CLAD diagnosis). The applied BAL protocol was in accordance with the recently published consensus statement for the standardization of BAL in LTx (20) (**Supplementary methods**).

### Peripheral Blood Collection

Peripheral blood samples were collected at the time of bronchoscopy at initial CLAD diagnosis, or if unavailable, the first available sample following CLAD diagnosis. (BD Vacutainer^®^ EDTA-lined plastic tubes, Franklin Lakes, NJ, USA). For stable LTx recipients, samples from the routine 2 years post-LTx visit were included. Blood plasma was isolated and stored at -80°C. As CTGF has a predominant renal clearance (21), plasma creatinine levels and the estimated glomerular filtration rate (eGFR - Chronic Kidney Disease Epidemiology Collaboration formula) were compared between groups as surrogate for potential differences in renal CTGF clearance.

### CTGF Enzyme-Linked Immunosorbent Assay

CTGF was determined by sandwich enzyme-linked immunosorbent assay (ELISA) as described previously (22, 23), using monoclonal antibodies against two distinct epitopes on the NH2-terminal part of human CTGF (FibroGen, South San Francisco, CA, USA). Twenty µl of undiluted BAL supernatants or 50 µl 1:5 diluted plasma were added to each well together with an equal volume of CTGF detection antibody conjugated to alkaline phosphatase. Purified recombinant human N-CTGF (FibroGen, South San Francisco, CA, USA) was used for calibration and absorbance was read at 405 nm. Measurements were performed in duplicate and the mean of these values was used for further analysis.

### Statistics and Ethics Statement

Results are presented as median and interquartile range (IQR), unless stated otherwise. All datasets were formally tested for normality using D’Agostino Pearson test. Discrete data were compared *via* contingency tables and Chi-square test. For continuous data, Mann-Whitney U and Kruskall-Wallis test was used with post-hoc testing using Dunn’s multiple comparison test, where appropriate. GraphPad prism version 8.0 software (San Diego, CA, USA) was used for all analyses. A two-tailed p-value of <0.05 was considered significant. This retrospective study was approved by the Leuven University Hospital institutional review board (S52174, S57742, S51577, S61653, S59648, ML6385) and performed according to the principles of the Declaration of Helsinki. Written informed consent to participate in biobanking and scientific research was provided by all CLAD and pulmonary GVHD patients included in the study. In addition, Belgian law states that donor organs not suitable for transplantation can be used in approved research programs.

## Results

### CTGF Explant Tissue Analysis

An overview of the patient characteristics of BOS, RAS, and control lungs included in the qPCR and immunohistochemical CTGF analysis is provided in **Tables S1** and **S2** *, respectively*. Control lungs (n=20) consisted of lungs not used for LTx for the following reasons: logistical issue (n=8), used for single/lobar transplant (n=7), extra-pulmonary tumor (n=3), and other (n=2). The cause of death of these donors was cerebrovascular accident (n=6), trauma (n=4), suicide (n=3), cardiac arrest (n=3), euthanasia (n=1), death during surgery (n=1), subarachnoid hemorrhage (n=1), and bacterial meningitis (n=1). Explant lung tissue from 9 patients with pulmonary GVHD was available (patient characteristics are provided in **Table S3**). Five (56%) of 9 patients suffered from an obstructive lung function deficit without prominent opacities on CT (i.e. obstructive pulmonary GVHD or BOS), while 4 (44%) patients suffered from a restrictive lung function deficit with ground glass opacities and consolidation on chest CT (i.e. restrictive pulmonary GVHD or late-onset non-infectious pulmonary complication other than BOS). An overview of the results of qPCR analysis, immunohistochemistry, BAL and blood plasma ELISA is provided in **Table S4**.

### qPCR Analysis

**Figure 1:**
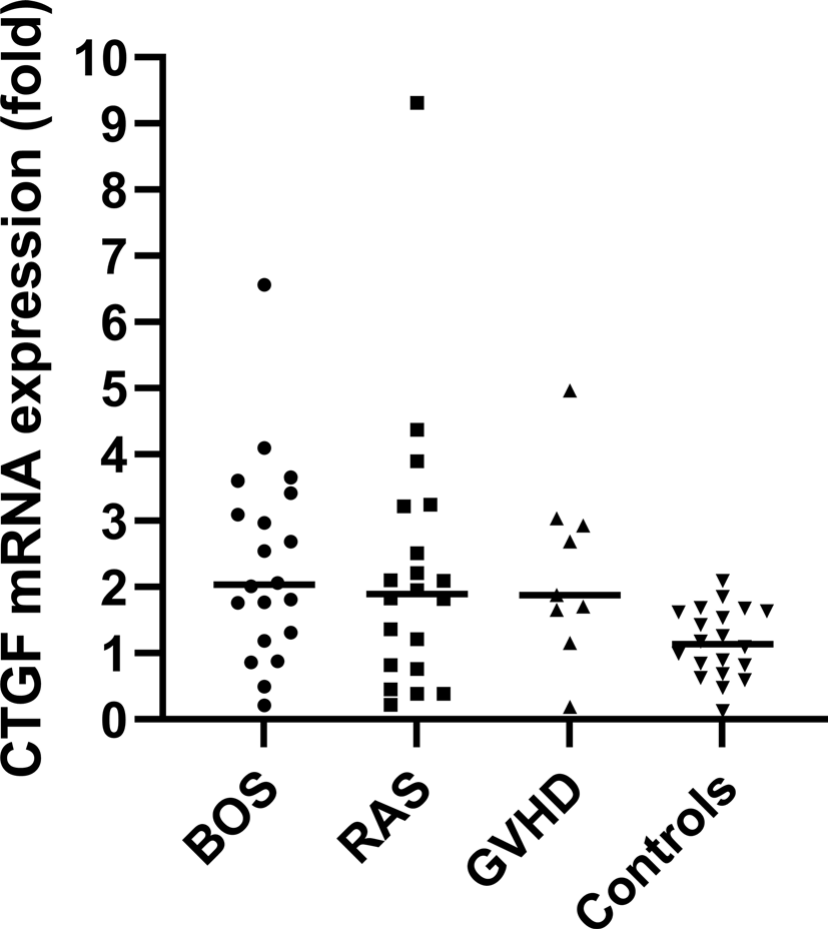
qPCR analysis with visualization of the difference in relative fold change levels of CTGF mRNA, relative to the geometric mean of the two housekeeping genes (TBP, GAPDH). Overall, CTGF levels were significantly increased compared to controls (Kruskal Wallis: p=0.014). *Post-hoc* analysis revealed significant increased relative CTGF mRNA levels in BOS *vs.* controls (p=0.0020) and GVHD vs. controls (p=0.0097).

qPCR analysis of BOS, RAS, pulmonary GVHD and control lungs revealed an overall significant difference in the relative fold change expression of CTGF mRNA compared to controls, normalized to the geometric mean of the two housekeeping genes (TBP, GAPDH) (p=0.014) (**Figure 1**). Post-hoc analysis revealed significant increased relative CTGF mRNA levels in BOS vs. controls (p=0.0020), GVHD vs. controls (p=0.0097), and trend towards higher CTGF mRNA content in RAS vs. controls (p=0.052).

### CTGF Staining in CLAD Explant Tissue

**Figure 2:**
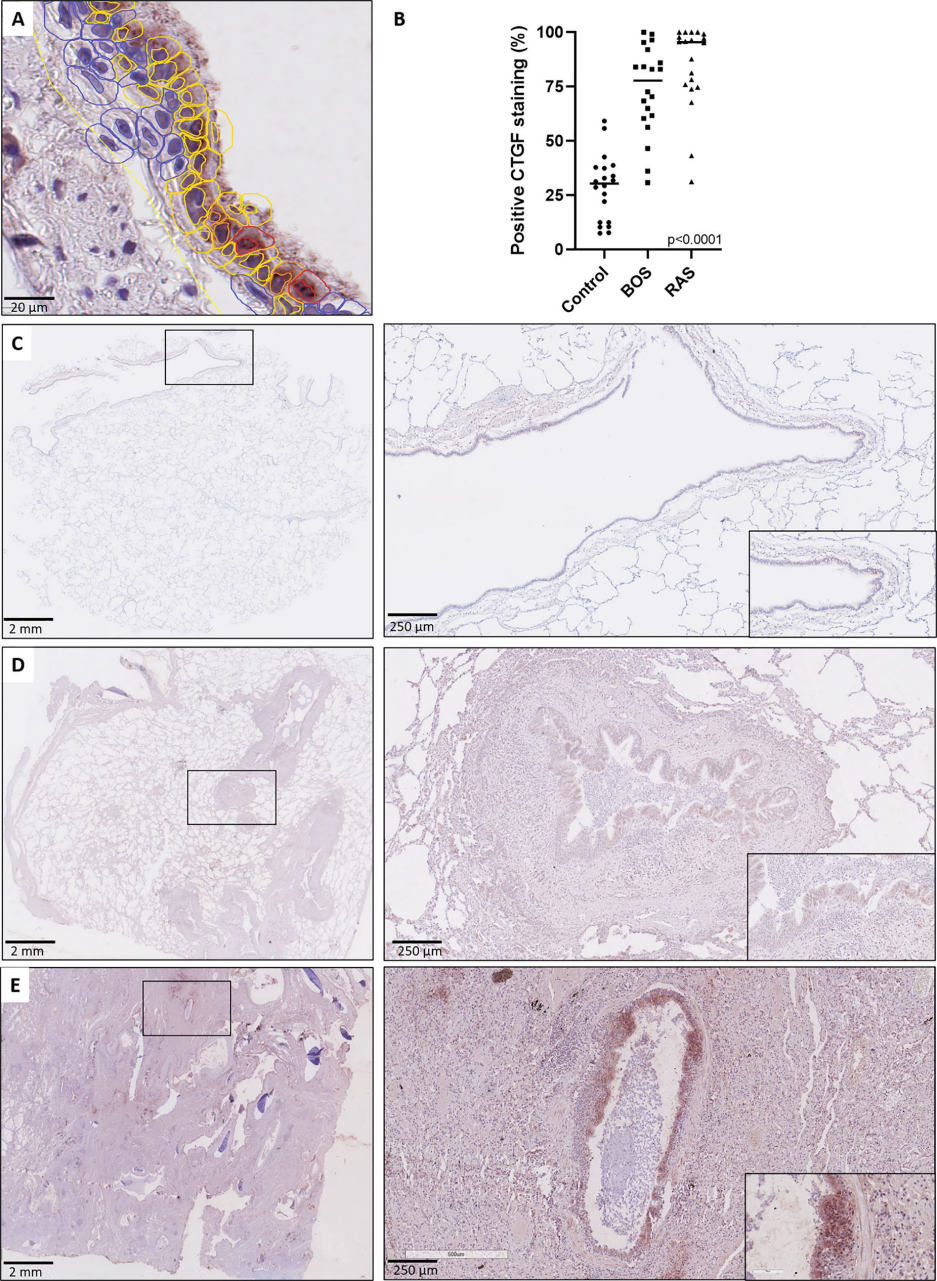
CTGF staining in respiratory epithelium. **(A)** High-detailed illustration of semi-automated cell segmentation and analysis method using QuPath software to assess the respiratory epithelium. Blue lining indicates no CTGF positive staining, yellow indicates mild (1+) staining, orange indicates moderate (2+) staining and red indicates strongly positive (3+) staining. **(B)** Comparison of CTGF immunohistochemistry revealed a significant higher percentage of CTGF-positive respiratory cells (staining intensity ≥1+) in both BOS and RAS, compared to control lungs (p<0.0001). **(C)** Control lung, normal lung parenchyma with presence of a larger normal airway. Inset: higher magnification of the normal lining respiratory epithelium, with presence of only few cells with a positive CTGF staining. **(D)** Bronchiolitis obliterans syndrome with normally preserved alveoli and the presence of several larger airways displaying mild to moderate sub-epithelial fibrosis. Inset: higher magnification of airway with diffuse cytoplasmic CTGF staining of the respiratory epithelium. **(E)** Restrictive allograft syndrome with severe fibrotic remodeling. Inset: airway with prominent and diffuse CTGF positive respiratory epithelium.

To validate and further explore the overall higher CTGF mRNA levels compared to control lungs, CTGF immunohistochemistry was performed to assess and compare protein expression patterns. Immunohistochemistry revealed a significant higher percentage of CTGF-positive respiratory epithelial cells (staining intensity ≥ 1+) in both end-stage BOS and RAS lungs (median: 77.81% [60.73-90.52], and median: 95.53%, [75.05-98.84]; respectively), compared to control lungs (median: 30.34%, [12.55-37.75], p<0.0001); while there was no significant difference between BOS and RAS (p=0.47) (**Figure 2** and comparison of different staining intensities is provided in **Table S4**).

**Figure 3:**
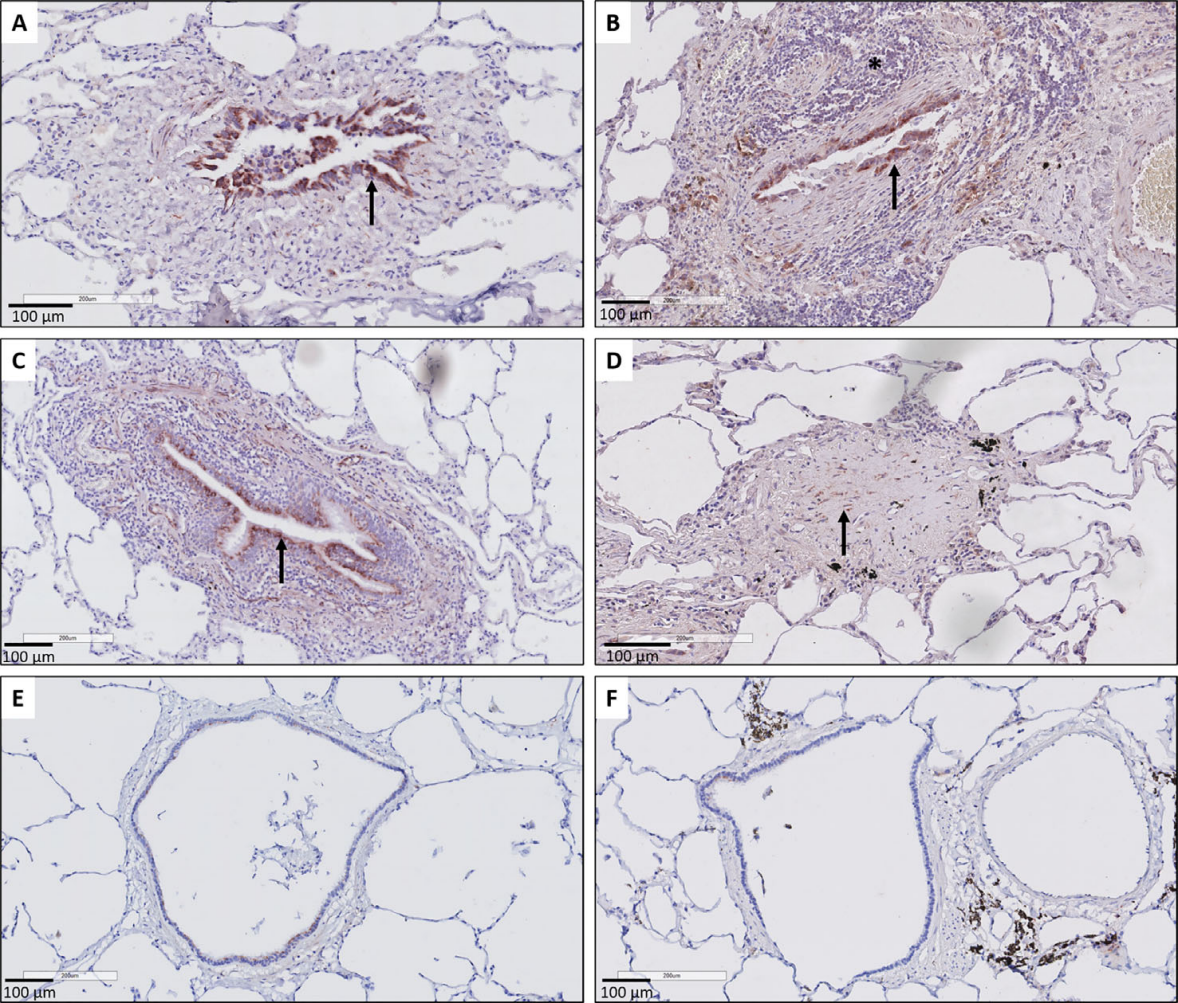
Representative immunohistochemical illustrations of CTGF staining in airway lesions in CLAD versus in normal bronchioles from control lungs. **(A, B)** BO lesions, characterized by sup-epithelial fibrosis are lined by respiratory epithelium with a diffuse and prominent CTGF positivity (arrows). In figure **(B)** there is also an accompanying prominent lymphocytic inflammation (asterisk). **(C)** Lymphocytic bronchiolitis characterized by diffuse submucosal invasion of lymphocytes with diffuse and strong CTGF positive stained respiratory epithelium (arrow). **(D)** end-stage BO lesion without recognizable airway lumen, with partly CTGF positive stromal cells (arrow). **(E, F)** bronchioles from control lungs, approximately the same size as the airways with obliterative airway remodeling in CLAD, displaying only limited and focal CTGF positive staining of the respiratory epithelium. In figure **(F)** there is also prominent presence of black pigment-laden macrophages.

Histopathological examination further revealed that the respiratory epithelium in active BO lesions, which are characterized by sub-epithelial fibrosis and potential inflammation of the small bronchioles, displayed a uniform prominent cytoplasmic CTGF staining (**Figures 3A, B**). Similarly, there was prominent staining in bronchioles with lymphocytic bronchiolitis (**Figure 3C**). In end-stage BO lesions, the respiratory epithelium was no longer discernable and only fibrotic lesions, with CTGF positive stromal cells, remained (**Figure 3D**). In contrast, normal bronchioles from the same size revealed only mild and limited focal CTGF staining of the respiratory epithelium in control lungs (**Figures 3E, F**).

**Figure 4:**
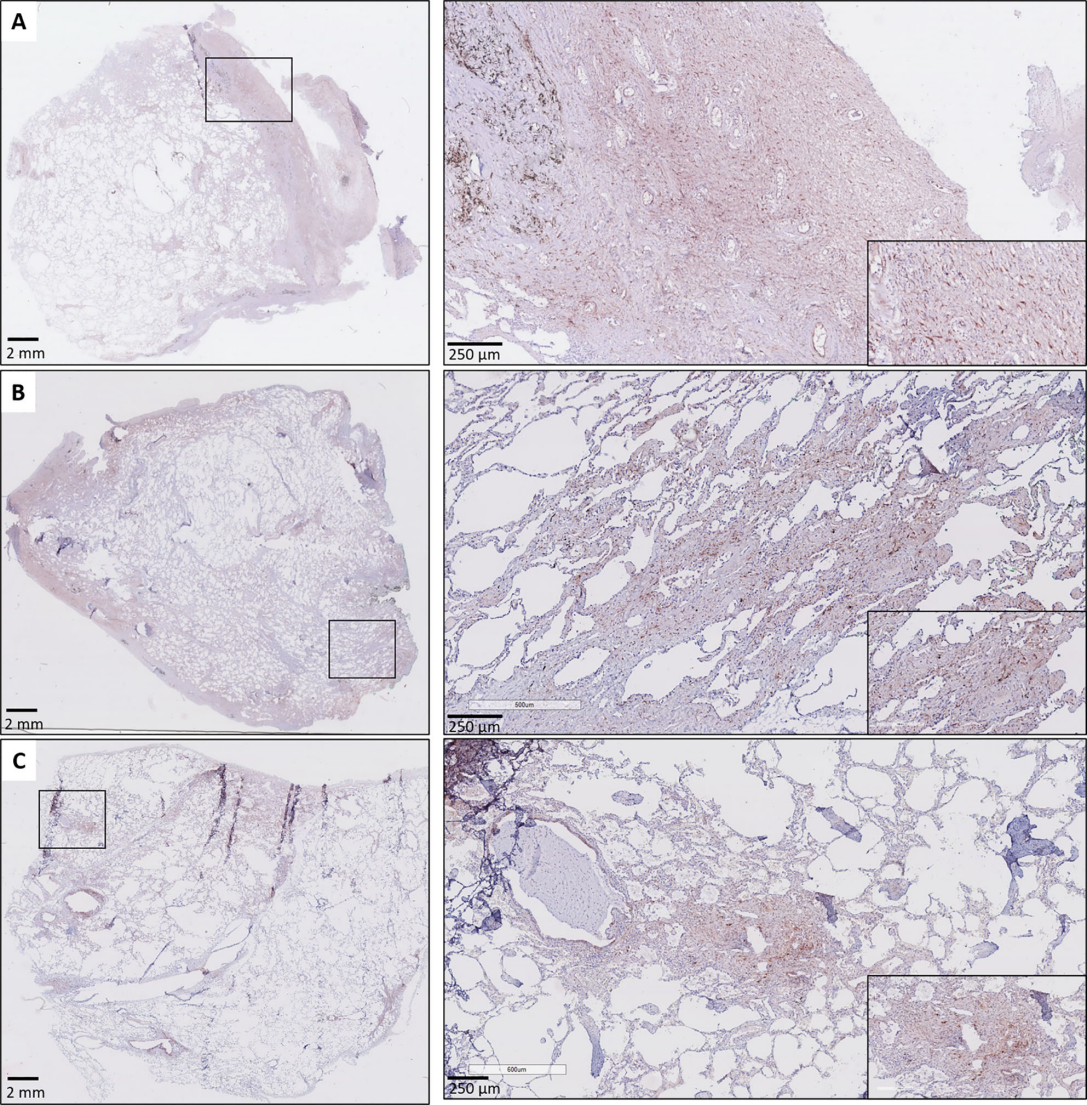
Fibrosis in CLAD. **(A)** Subpleural fibrosis in a patient with RAS. The inset illustrates prominent and diffuse (myo)fibroblast staining in the (sub)pleural region. **(B)** RAS, with both subpleural fibrosis and alveolar fibrosis. The inset illustrates prominent CTGF staining in a zone with thickened fibrotic alveolar septa. **(C)** BOS. The lung parenchyma has signs of mild emphysema, but no obvious fibrotic remodeling is present. The inset highlights a microscopic focus of fibrosis, in which there is also prominent CTGF staining of fibroblasts.

**Figure 5:**
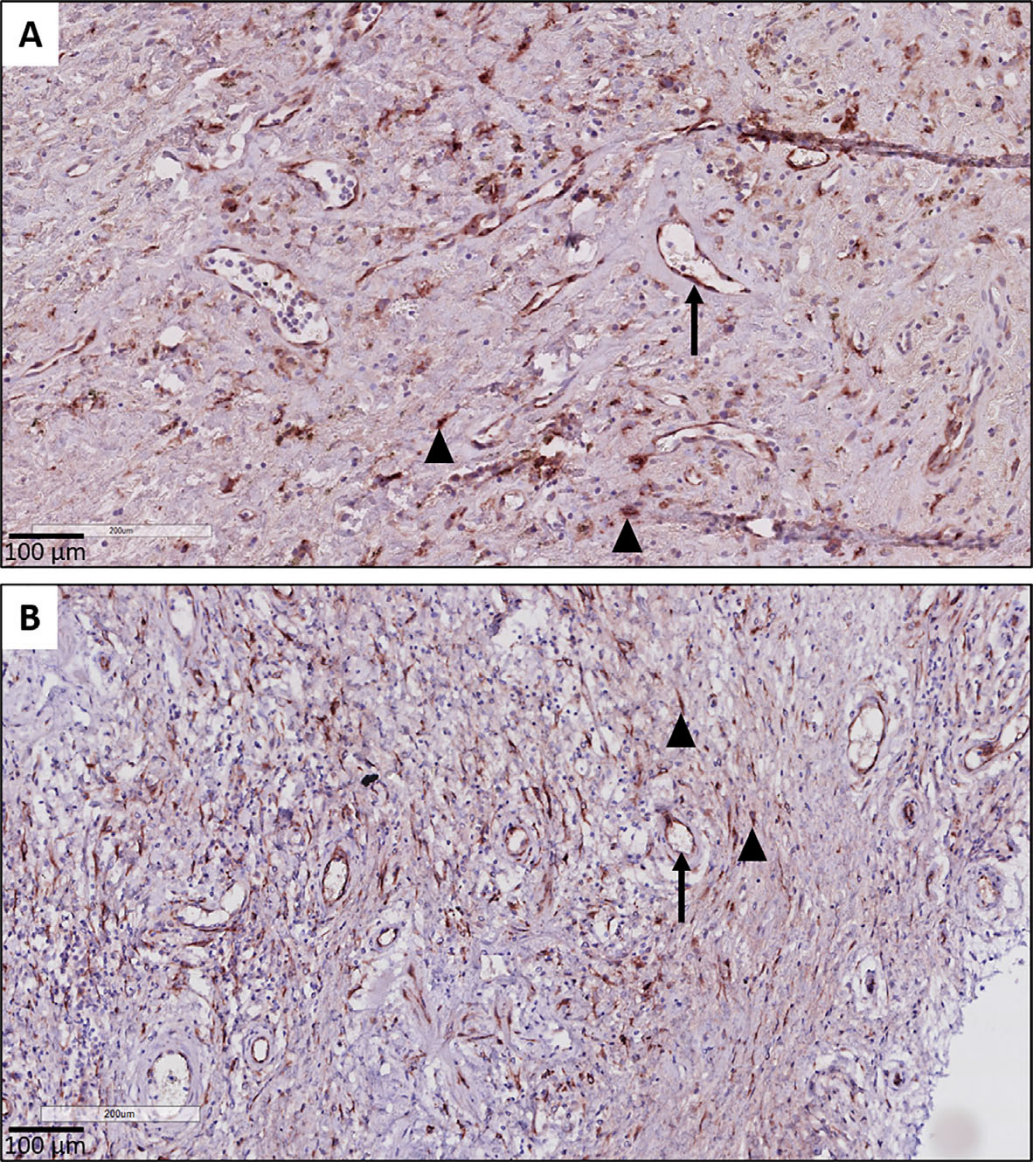
CTGF staining in endothelial cells. **(A, B)** Illustration of prominent endothelial staining (arrows) in fibrotic zones of two patients with RAS. The (myo)fibroblasts are also strongly CTGF positive (arrow heads).

A consistent finding was the presence of strongly positive CTGF stained stromal fibroblasts in fibrotic regions of RAS lungs. This included fibrosis in regions of (sub)pleural fibrosis and alveolar fibrosis (e.g. diffuse fibrotic thickening of alveolar septa) (**Figures 4A, B**, respectively). In addition, microscopic interstitial foci of fibrosis in BOS lungs, beyond the fibrosis in the setting of (end-stage) BO lesions, displayed a comparable and consistent strongly positive staining of CTGF positive fibroblasts (**Figure 4C**). CTGF immunohistochemical staining of endothelial cells was variable, but consistently prominently expressed in fibrotic regions of RAS patients (**Figures 5A, B**). CTGF staining of macrophages also displayed considerable variation in staining intensities. The most prominent finding was CTGF positive staining of collections of intra-alveolar macrophages, which were most abundant in RAS and typically distributed around zones of interstitial fibrosis (**Supplementary Figure S2**). However, even within and between these collections of intra-alveolar macrophages, staining intensity varied.

### CTGF Expression in GVHD

**Figure 6:**
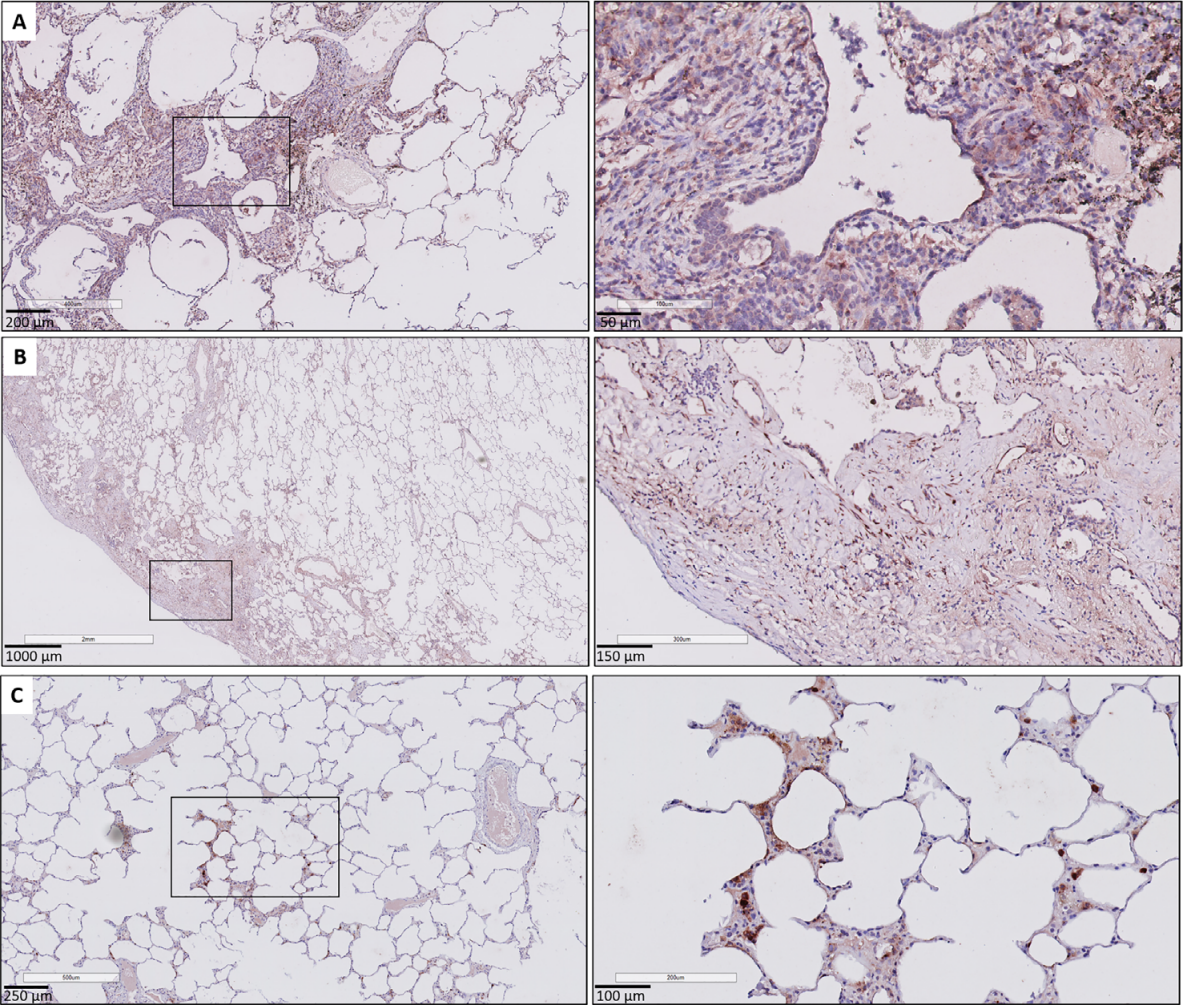
CTGF immunohistochemistry in patients with pulmonary GVHD after HCT. **(A)** Illustration of small bronchiole surrounded by fibrous remodeling with limited inflammation, with CTFG positive respiratory epithelium and stromal cells. **(B)** Presence of subpleural fibrosis characterized by prominent staining of the (myo)fibroblasts in these fibrotic regions. **(C)** Presence of focal, minimal fibrous thickening of the alveolar septa, with presence of prominent staining of interstitial macrophages and the alveolar epithelial cells. The surrounding alveolar parenchyma is normally preserved.

Immunohistochemical CTGF analysis of GVHD lung tissue revealed similar histological findings compared to BOS and RAS. GVHD lungs displayed prominent staining intensity (≥ 1+) of the respiratory epithelium (median percentage of positive cells: 88.87% [83.13-91.64]), significantly increased compared to unused donor lungs (p<0.0001). Similar to CLAD, a diffuse cytoplasmic CTGF positivity of the respiratory epithelium in small bronchioles with fibrotic remodeling was found (**Figure 6A**). In fibrotic zones, similar to RAS, there was strong and diffuse CTGF staining of the fibroblasts (**Figures 6B, C**). Endothelial cells also displayed variable CTGF staining, but, similar as in RAS, were consistently highly expressed in fibrotic regions.

### CTGF Biomarker Exploration

Since end-stage explant lung tissue analysis revealed higher CTGF expression in CLAD compared to control lungs, with prominent CTGF expression in the respiratory epithelium and endothelium, we aimed to explore the role of CTGF as a potential biomarker for CLAD, based on CTGF levels in BAL and plasma. Patient characteristics of BOS (n=20), RAS (n=20) and stable LTx (n=20) recipients included for BAL and plasma CTGF analysis are provided in **Table S5**. For pulmonary GVHD patients, no BAL and plasma samples were available. Patients had a median time to BOS of 3.10 years [1.46-4.58] and to RAS of 3.38 years [1.81-4.60]. Graft loss occurred in 6/20 (30%) BOS patients and 11/20 (55%) RAS patients. In addition, graft loss occurred in 2/20 (10%) patients from the stable group (i.e. 9 and 10 years after LTx due to hospital acquired pneumonia following orthopedic surgery, and sudden cardiac arrest, respectively), without available explant tissue.

### BAL CTGF ELISA

**Figure 7:**
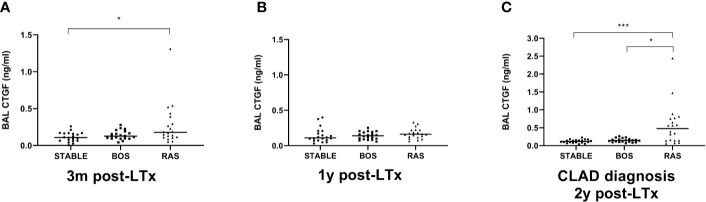
Comparison of BAL CTGF protein levels between groups at different time points. **(A)** CTGF values at 3 months after LTx (p=0.028). CTGF values are significantly higher in patients that later developed RAS, compared to stable LTx recipients (p=0.025). **(B)** CTGF values at 1 year after LTx, there are no significant differences between groups (p=0.20). **(C)** CTGF values at CLAD diagnosis and at 2 years post-LTx for stable LTx recipients (p=0.0009). CTGF values are significantly higher at RAS diagnosis compared to stable LTx recipients at 2 years post LTx (p=0.0007) and compared to CTGF values at BOS diagnosis (p=0.042). BAL, broncho-alveolar lavage; BOS, bronchiolitis obliterans syndrome; RAS, restrictive allograft syndrome; CLAD, chronic lung allograft dysfunction.

**Figure 8:**
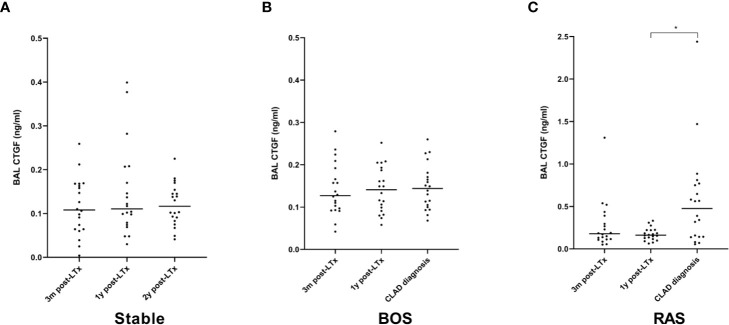
Paired analysis of BAL CTGF protein levels at the different time points after LTx. **(A)** CTGF values for stable LTx recipients; there was no significant difference between different time points (p=0.84). **(B)** CTGF values for BOS patients; no significant difference was observed between different time points (p=0.92). **(C)** CTGF values for RAS patients; CTGF values were significantly higher at CLAD diagnosis (p=0.026); *post-hoc* analysis confirmed higher CTGF values at CLAD diagnosis compared to 1y post-LTx (p=0.029), but not compared to 3 months post-LTx (p=0.15). BOS, bronchiolitis obliterans syndrome; RAS, restrictive allograft syndrome.

BAL CTGF content was assessed at three distinct time points: at 3 months post-LTx, 1 year post-LTx, and at CLAD diagnosis or at 2 years post-LTx in stable LTx recipients. CTGF levels were significantly higher at 3 months post-LTx in patients who later developed RAS compared to stable LTx recipients (p=0.025), while there was no significant difference with patients who later developed BOS (p=0.28) (**Figure 7A**). At 1 year post-LTx, there was no significant difference between groups (p=0.20) (**Figure 7B**). At CLAD diagnosis, CTGF levels were significantly higher in RAS patients compared to stable LTx recipients (p=0.0007) and BOS patients (p=0.042) (**Figure 7C**). Paired analysis revealed no significant difference in CTGF values at the different time points for stable and BOS patients (p=0.84 and p=0.92, respectively) (**Figures 8A, B**), whereas values had significantly increased in RAS patients at the moment of CLAD diagnosis compared to 1 year post-LTx (p=0.029) (**Figure 8C** and **Supplementary Figure S3**).

### Plasma CTGF ELISA

**Figure 9:**
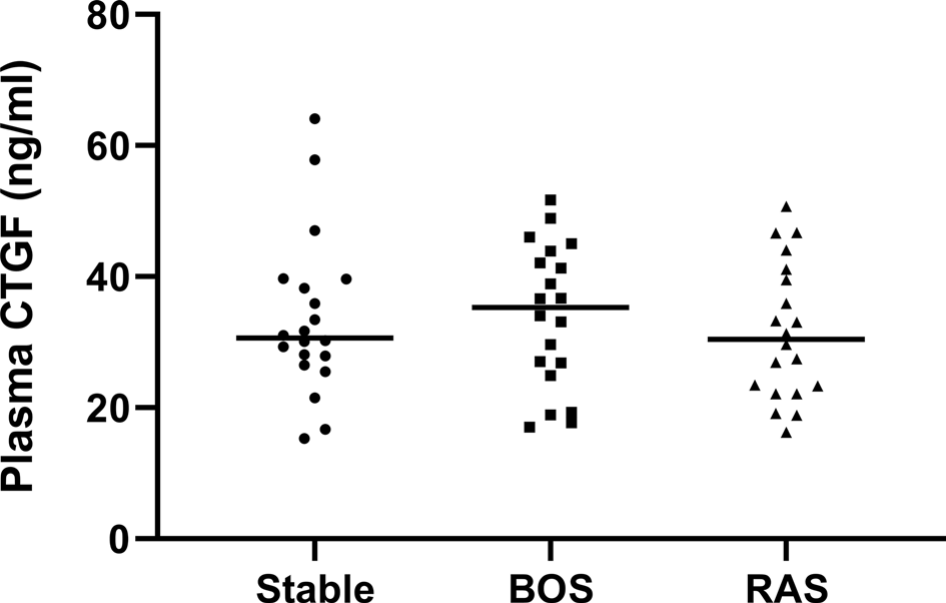
Plasma CTGF protein levels. There was no significant difference in plasma CTGF levels between groups (p=0.74).

Plasma CTGF analysis from BOS, RAS, and stable LTx patients (obtained at routine 2 years post-LTx visit) revealed no significant differences (p=0.74) (**Figure 9**). Blood samples were collected at initial BOS diagnosis for 3/20 (15%) BOS patients, and at initial RAS diagnosis for 12/20 (60%) RAS patients, while the remaining samples were obtained after initial CLAD diagnosis (median time after CLAD diagnosis was 2.30 years [0.89-4.92]). Median plasma creatinine levels and the estimated glomerular filtration rate did not significantly differ between groups, indicating no difference in renal function and therefore presumably no difference in renal CTGF clearance (p=0.95 and p=0.80, respectively) (**Table S4**).

## Discussion

This explorative study investigated the expression of CTGF in CLAD and pulmonary GVHD. The main findings of this study are (i) higher levels of tissue CTGF expression in end-stage explant lungs of BOS, RAS and pulmonary GVHD patients compared to control lungs and (ii) increased CTGF levels in BAL at RAS diagnosis compared to stable LTx patients and compared to BOS patients.

CTGF has an important role in many biological processes, such as cell adhesion, proliferation, differentiation, and tissue repair. It stands as a central profibrotic mediator, and its transcription is regulated by several factors, both directly from external stimuli (e.g. hypoxia, mechanical stimulation) and through cross-talks (e.g. TGF-β) (24). CTGF has been studied in several organs, including kidney, liver, cardiac and lung fibrosis (1). In IPF, CTGF was found to be upregulated in cultured fibroblasts, broncho-alveolar lavage, plasma, and lung tissue (25–28). In animal models, CTGF levels were increased in fibroblasts of a bleomycin-induced mouse model of IPF (29), and CTGF was an essential factor to induce a profibrotic environment in “fibrosis-resistant” mice lungs (30). These findings in the field of IPF, combined with overt fibrotic lung remodeling in CLAD and pulmonary GVHD, therefore also provide a theoretical basis for studying the role of CTGF in CLAD. However, in the context of CLAD after LTx or pulmonary GVHD after HCT, no data are currently available regarding CTGF expression. To the best of our knowledge, this study therefore forms the first to explore the role of CTGF in CLAD and pulmonary GVHD.

Exploration of CTGF expression in explant lungs revealed prominent differences between CLAD (BOS/RAS) and pulmonary GVHD on the one hand, and control lungs on the other hand. CTGF staining was significantly increased in the respiratory epithelium in CLAD and pulmonary GVHD compared to control lungs, with especially prominent staining of the respiratory epithelium in BO lesions. Interestingly, higher respiratory CTGF expression has previously been demonstrated in chronic obstructive pulmonary disease, and correlated with disease severity (31). Further, CLAD explant lungs displayed consistent and strong CTGF positivity in fibroblasts, comparable to previously described findings in IPF (28). In addition, CTGF expression in endothelial cells was specifically prominent in fibrotic zones of RAS patients. CTGF is known to exacerbate vascular remodeling in the lung, and CTGF transcription can be induced by hypoxia (1). In transgenic mouse models, down-regulation of CTGF was protective against development of pulmonary hypertension associated with hypoxia and against bleomycin-induced pulmonary hypertension (32). Modulation of endothelial CTGF expression may therefore be a potential interesting mechanism of action for CTGF-inhibitors, especially since pulmonary hypertension and vascular remodeling is highly prevalent in CLAD patients (33).

As immunohistochemical analysis revealed prominent CTGF expression in the respiratory epithelium and endothelium, monitoring of CTGF levels in BAL and plasma might hold potential as a biomarker. CTGF ELISA, mostly measured in plasma and urine, has previously been proposed as a biomarker monitoring tool to measure the extent of ongoing fibrosis in several fibrotic diseases (liver fibrosis, diabetic nephropathy, systemic sclerosis), and correlated with disease severity (1). Our current findings confirm that CTGF expression in BAL may be associated with fibrosis in CLAD, although the results were variable between patients, potentially due to the inherent sampling-variability observed in fibrotic remodeling in CLAD patients. In addition, we found no increased plasma CTGF expression in our cohort. While BAL CTGF was higher in RAS at 3 months post-LTx compared to stable LTx patients, this difference did not persist in consecutive samples at 1 year post-LTx. This lowers the current evidence of CTGF as potential biomarker to predict CLAD onset and advocates further investigation of CTGF biomarker potential in successive BAL samples of a larger cohort. However, it is important to note that a BAL procedure gives no information about the whole lung, but rather for a limited bronchopulmonary segment. As fibrotic remodeling in CLAD is a heterogeneous phenomenon, especially given the preferential apical fibrosis in RAS, BAL fluid analysis might underestimate the true observed difference. Moreover, BAL samples were obtained at CLAD diagnosis, when fibrotic remodeling may still be limited.

Exploration of potential biomarkers to predict CLAD development has been performed by several groups (34). Despite the fact that no single clinically useful biomarker has been identified yet, several interesting markers have been linked to CLAD development in BAL, blood and lung tissue. Todd et al. recently identified upregulation of amphiregulin, an epidermal growth factor receptor (EGFR) ligand with putative role in airway injury and repair regulation, in BAL fluid of CLAD patients (20). *In situ* hybridization of CLAD tissue identified abundant EGFR and amphiregulin transcript expression in the airway epithelial cells of fibrotic regions. Moreover, *ex vivo* stimulation of cultured bronchial epithelial cells with amphiregulin led to increased hyaluronan expression through an EGRF-dependent pathway. Hyaluronan is an important glycosaminoglycan previously found to be increased in both BAL and blood of CLAD patients (35). In addition, a murine orthotopic lung transplant model demonstrated that hyaluronan might contribute to CLAD by activation of innate immune signaling pathways in a toll-like receptor (TLR) 2/4 and myeloid differentiation primary response (MyD)-88 dependent manner (35). Shino et al. analyzed BAL expression of Chemokine (C-X-C motif) ligand (CXCL) 9, CXCL10 and CXCL11, which form CXC chemokine receptor (CXCR) 3 chemokine ligands and which act as chemoattractants of mononuclear cells. They found, in a multivariate adjusted model that higher expression of these CXCR3 chemokine ligands in BAL, in co-presence with organizing pneumonia, led to significantly increased CLAD risk (36). Very recently, the same group reported increased BAL levels of CXCL9 and CXCL10 in a prospective multicenter study during episodes of acute rejection and acute lung injury in the first year post-LTx, which form known risk factors for future CLAD development (37).

Micro-array based gene expression analysis of BAL cell pellets by Weigt et al., obtained at one year post transplant surveillance in a small nested-case control study, revealed differential gene expression related to mainly CD8 cytotoxic lymphocytes and NK cell activation and proliferation in patients who developed CLAD within two years after bronchoscopy (38). Micro-array analysis of blood samples however mostly pointed towards a qualitative B cell defect, with identification of a three gene based predictive model of CLAD (downregulation of POU class 2 associating factor 1 (POU2AF1), T cell leukemia/lymphoma protein 1A (TCL1A) and B cell lymphocyte kinase (BLK)) (39). In blood, especially MMP-9 has further gained interest as a potential biomarker. Pain et al. reported that increased expression of MMP-9 was independently associated with CLAD and detection preceded functional CLAD diagnosis by 12 months (40). Interestingly, in an associated *ex vivo* study, they identified that activated T cells promote specific MMP‐9 production by bronchial epithelial cells trough the CCL2/CCR2 axis in synergy with TGF‐β and initiate epithelial-to-mesenchymal transition (40). Recently, increased expression of lipocalin-2, which stabilizes MMP-9 activity, was also proposed as potential biomarker to distinguish RAS from BOS patients and stable-LTx patients (41). TGF-β forms the most potent inducer of CTGF, and CTGF overproduction plays a crucial role in fibrosis development (42, 43), but appears to induce fibrosis mostly in the co-presence of TGF-β (44). We previously reported that TGF-β was mainly upregulated in RAS patients, both in BAL fluid and explant lung tissue, and TGF-β stimulation of pleural mesothelial cells led to a phenotypical switch to mesenchymal cells, indicating a potential role in the epithelial-to-mesenchymal transition (45).

In a molecular profiling study based on lung biopsies obtained in the first year post-LTx, Jonigk et al. assessed 45 tissue remodeling-associated genes in a cohort that rapidly developed CLAD within 3y post-LTx versus a matched stable cohort without CLAD development (8). By combining mRNA expression levels of the five most significantly differentially expressed genes from the TGF-β axis (BMP-4, IL-6, MMP-1, SMAD1, and THBS1), they could predict patient outcomes. In addition, the same group performed laser-assisted microdissection of airway lesions in both CLAD and IPF explant lungs, and reported a large and important molecular overlap of pivotal fibrotic pathways, with shared upregulation of TGF-β, MMP-9, RANTES, TIMP-1, TIMP-2, BMP-2, PLA-1 and COL1/2/3 (6).

There is currently no effective treatment for CLAD, and especially RAS patients typically experience a rapid inevitable decline of pulmonary function after diagnosis, with a median survival of only 1-1.5 years (9). Given the clinical, radiological and pathological similarities with IPF, attempted treatment strategies are mainly based on approved IPF treatments. Pirfenidone appears safe and may attenuate the rate of decline in lung function in patients with RAS, although prospective trials are lacking (46, 47). In BOS patients, both Pirfenidone and Nintedanib, another approved IPF treatment, are currently investigated in prospective clinical trials (NCT02262299 and NCT03283007, respectively). CTGF has also gained interest as a potential therapeutic target for IPF. A clinical phase II placebo-controlled trial of a fully recombinant human monoclonal antibody against CTGF (FG-3019) was recently found to attenuate progression of IPF (2). Despite that the phase III trial is currently still ongoing, this might perhaps become a third therapeutic option for IPF in the near future (NCT03955146). Given the striking similarities between IPF and CLAD, and given our current findings, this might also indirectly advocate to study CTGF as potential therapeutic target in CLAD, and by extrapolation, in pulmonary GVHD.

There are some limitations to our study. First, this is an explorative and descriptive retrospective single center study with limited sample size and although patients were well-characterized, extrapolation of these results might therefore be limited. Second, serum samples at CLAD diagnosis were not available for most CLAD patients due to the fact that serum samples were only routinely obtained from 2015 onwards. Further exploration of the biomarker potential of CTGF ELISA remains to be investigated in a larger cohort. Third, no lung tissue was available from stable LTx recipients due to inherent limitations in collecting explant material and no immunohistochemical comparison could therefore be made between CLAD and stable LTx patients. However, matched unused donor lungs served as a valuable and unique control group. Finally, no BAL or blood samples were available from pulmonary GVHD patients, as well as only a limited number of lungs, which is directly related to the low number of GVHD patients qualifying for LTx and the limited number of LTx for this indication in most centers.

In conclusion, lung tissue CTGF expression levels are mainly increased in end-stage RAS, but also in established BOS and pulmonary GVHD. CTGF protein expression is increased in BAL fluid from RAS patients at CLAD diagnosis compared to BOS and stable LTx patients. Our results therefore suggest a potential role for CTGF in CLAD, especially RAS, and pulmonary GvHD, which advocates further investigation of the role of CTGF in the pathophysiology of CLAD and the potential therapeutic modulation of CTGF in onset and progression of both disorders.

## Data Availability Statement

The raw data supporting the conclusions of this article will be made available by the authors, without undue reservation.

## Ethics Statement

The studies involving human participants were reviewed and approved by UZ Leuven Ethical Committee. The patients/participants provided their written informed consent to participate in this study.

## Author Contributions

Concept and design: AV, RG, RB, TN, AS, SV, BW, and RV. Data analysis and interpretation: AV, RG, RB, TN, AS, JK, TH, SV, AZ, GV, BW, EV, LC, DR, AN, HS, BV, and RV. Drafting the manuscript for important intellectual content: AV, RG, RB, TN, AS, JK, TH, SV, AZ, GV, BW, EV, LC, DR, AN, HS, BV, and RV. All authors contributed to the article and approved the submitted version.

## Funding

AV is sponsored by a fundamental research grant from the FWO (1102020N). JK is sponsored by a fundamental research grant from the FWO (1198920N). SV is sponsored by a grant from the FWO (FWO12G8715N) and the KU Leuven (C24/18/073). BV is funded by the KU Leuven (C24/050). RV is supported by the FWO (senior clinical researcher) and by a Roche Research Grant from the Belgian Transplant Society. LC is supported by a KU Leuven University chair sponsored by the company Medtronic. DR and GV are supported by the Broere Charitable foundation.

## Conflict of Interest

RG has received research support from Fibrogen Inc.

The remaining authors declare that the research was conducted in the absence of any commercial or financial relationships that could be construed as a potential conflict of interest.

## Abbreviations

BAL, broncho-alveolar lavage; BOS, bronchiolitis obliterans syndrome; CKD-EPI, Chronic Kidney Disease Epidemiology Collaboration formula; CLAD, chronic lung allograft dysfunction; CT, computed tomography; CTGF, connective tissue growth factor; eGFR, estimated glomerular filtration rate; FEV1, forced expiratory volume in one second; FFPE, formalin-fixed and paraffin-embedded; FVC, forced vital capacity; GVHD, graft-versus-host-disease; HCT, hematopoietic stem cell transplantation; IPF, idiopathic pulmonary fibrosis; LTx, lung transplantation; MMP, matrix metalloproteinases; qPCR, quantitative real-time polymerase chain reaction; RAS, restrictive allograft syndrome; TGF-β, transforming growth factor beta; TIMP, tissue inhibitors of metalloproteinases; TLC, total lung capacity.

## References

[1] Ramazani Y Knops N Elmonem MA Nguyen TQ Arcolino FO van den Heuvel L. Connective Tissue Growth Factor (CTGF) From Basics to Clinics. Matrix Biol (2018) 68–69:44–66. 10.1016/j.matbio.2018.03.007 29574063

[2] Richeldi L Fernández Pérez ER Costabel U Albera C Lederer DJ Flaherty KR. Pamrevlumab, an Anti-Connective Tissue Growth Factor Therapy, for Idiopathic Pulmonary Fibrosis (PRAISE): A Phase 2, Randomised, Double-Blind, Placebo-Controlled Trial. Lancet Respir Med (2020) 8(1):25–33. 10.1016/S2213-2600(19)30262-0 31575509

[3] Chambers DC Yusen RD Cherikh WS Goldfarb SB Kucheryavaya AY Khusch K. The Registry of the International Society for Heart and Lung Transplantation: Thirty-Fourth Adult Lung and Heart-Lung Transplantation Report—2017; Focus Theme: Allograft Ischemic Time. J Heart Lung Transplant (2017) 36(10):1047–59. 10.1016/j.healun.2017.07.016 28784324

[4] Kulkarni HS Cherikh WS Chambers DC Garcia VC Hachem RR Kreisel D. Bronchiolitis Obliterans Syndrome–Free Survival After Lung Transplantation: An International Society for Heart and Lung Transplantation Thoracic Transplant Registry Analysis. J Heart Lung Transplant (2019) 38(1):5–16. 10.1016/j.healun.2018.09.016 30391193 PMC6431291

[5] Verleden GM Glanville AR Lease ED Fisher AJ Calabrese F Corris PA. Chronic Lung Allograft Dysfunction: Definition, Diagnostic Criteria, and Approaches to Treatment―a Consensus Report From the Pulmonary Council of the ISHLT. J Heart Lung Transplant (2019) 38(5):493–503. 10.1016/j.healun.2019.03.009 30962148

[6] Jonigk D Merk M Hussein K Maegel L Theophile K Muth M. Obliterative Airway Remodeling Molecular Evidence for Shared Pathways in Transplanted and Native Lungs. Am J Pathol (2011) 178(2):599–608. 10.1016/j.ajpath.2010.10.032 21281792 PMC3070560

[7] Kuehnel M Maegel L Vogel-Claussen J Robertus JL Jonigk D. Airway Remodelling in the Transplanted Lung. Cell Tissue Res (2017) 367(3):663–75. 10.1007/s00441-016-2529-0 27837271

[8] Jonigk D Izykowski N Rische J Braubach P Kühnel M Warnecke G. Molecular Profiling in Lung Biopsies of Human Pulmonary Allografts to Predict Chronic Lung Allograft Dysfunction. Am J Pathol (2015) 185(12):3178–88. 10.1016/j.ajpath.2015.08.016 26476349

[9] Sato M Waddell TK Wagnetz U Roberts HC Hwang DM Haroon A. Restrictive Allograft Syndrome (RAS): A Novel Form of Chronic Lung Allograft Dysfunction. J Heart Lung Transplant (2011) 30(7):735–42. 10.1016/j.healun.2011.01.712 21419659

[10] Glanville AR Verleden GM Todd JL Benden C Calabrese F Gottlieb J. Chronic Lung Allograft Dysfunction: Definition and Update of Restrictive Allograft Syndrome―a Consensus Report From the Pulmonary Council of the ISHLT. J Heart Lung Transplant (2019) 38(5):483–92. 10.1016/j.healun.2019.03.008 31027539

[11] Ofek E Sato M Saito T Wagnetz U Roberts HC Chaparro C. Restrictive Allograft Syndrome Post Lung Transplantation is Characterized by Pleuroparenchymal Fibroelastosis. Modern Pathol (2013) 26:350–6. 10.1038/modpathol.2012.171 23018877

[12] Palmer J Williams K Inamoto Y Chai X Martin PJ Tomas LS. Pulmonary Symptoms Measured by the National Institutes of Health Lung Score Predict Overall Survival, Nonrelapse Mortality, and Patient-Reported Outcomes in Chronic Graft-Versus-Host Disease. Biol Blood Marrow Transplant (2014) 20(3):337–44. 10.1016/j.bbmt.2013.11.025 PMC3973401 24315845

[13] Takeuchi Y Miyagawa-Hayashino A Chen F Kubo T Handa T Date H. Pleuroparenchymal Fibroelastosis and Non-Specific Interstitial Pneumonia: Frequent Pulmonary Sequelae of Haematopoietic Stem Cell Transplantation. Histopathology (2015) 66(4):536–44. 10.1111/his.12553 25234860

[14] Jonigk D Rath B Borchert P Braubach P Maegel L Izykowski N. Comparative Analysis of Morphological and Molecular Motifs in Bronchiolitis Obliterans and Alveolar Fibroelastosis After Lung and Stem Cell Transplantation. J Pathol Clin Res (2017) 3(1):17–28. 10.1002/cjp2.60 28138398 PMC5259562

[15] Jagasia MH Greinix HT Arora M Williams KM Wolff D Cowen EW. National Institutes of Health Consensus Development Project on Criteria for Clinical Trials in Chronic Graft-Versus-Host Disease: I. the 2014 Diagnosis and Staging Working Group Report. Biol Blood Marrow Transplant (2015) 21(3):389–401. 10.1016/j.bbmt.2015.02.025 25529383 PMC4329079

[16] Verleden SE Kirby M Everaerts S Vanstapel A McDonough JE Verbeken EK. Small Airway Loss in the Physiologically Ageing Lung: A Cross-Sectional Study in Unused Donor Lungs. Lancet Respir Med (2021) 9(2):167–174. 10.1016/S2213-2600(20)30324-6 33031747

[17] Falke LL Kinashi H Dendooven A Broekhuizen R Stoop R Joles JA. Age-Dependent Shifts in Renal Response to Injury Relate to Altered BMP6/CTGF Expression and Signaling. Am J Physiol Physiol (2016) 311(5):926–34. 10.1152/ajprenal.00324.2016 27558559

[18] Bankhead P Loughrey MB Fernández JA Dombrowski Y Mcart DG Dunne PD. Qupath: Open Source Software for Digital Pathology Image Analysis. Sci Rep (2017) 7(1):16878. 10.1038/s41598-017-17204-5 29203879 PMC5715110

[19] Vanstapel A Verleden SE Weynand B Verbeken E De Sadeleer L Vanaudenaerde BM. Late-Onset “Acute Fibrinous and Organising Pneumonia” Impairs Long-Term Lung Allograft Function and Survival. Eur Respir J (2020) 56(3):1902292. 10.1183/13993003.02292-2019 32381491

[20] Martinu T Koutsokera A Benden C Cantu E Chambers D Cypel M. Ishlt Consensus Statement for the Standardization of Bronchoalveolar Lavage in Lung Transplantation. J Heart Lung Transplant (2020) 39(11):1171–90. 10.1016/j.healun.2020.07.006 PMC7361106 32773322

[21] Gerritsen KG Abrahams AC Peters HP Nguyen TQ Koeners MP Den Hoedt CH. Effect of GFR on Plasma N-Terminal Connective Tissue Growth Factor (CTGF) Concentrations. Am J Kidney Dis (2012) 59(5):619–27. 10.1053/j.ajkd.2011.12.019 22342213

[22] Nguyen TQ Tarnow L Andersen S Hovind P Parving HH Goldschmeding R. Urinary Connective Tissue Growth Factor Excretion Correlates With Clinical Markers of Renal Disease in a Large Population of Type 1 Diabetic Patients With Diabetic Nephropathy. Diabetes Care (2006) 29(1):83–8. 10.2337/diacare.29.01.06.dc05-1670 16373901

[23] Murphy M Godson C Cannon S Kato S Mackenzie HS Martin F. Suppression Subtractive Hybridization Identifies High Glucose Levels as a Stimulus for Expression of Connective Tissue Growth Factor and Other Genes in Human Mesangial Cells. J Biol Chem (1999) 274(9):5830–4. 10.1074/jbc.274.9.5830 10026205

[24] Kubota S Takigawa M. Cellular and Molecular Actions of CCN2/CTGF and Its Role Under Physiological and Pathological Conditions. Clin Sci (2014) 128(3):181–96. 10.1042/CS20140264 25294165

[25] Plantier L Renaud H Respaud R Marchand-Adam S Crestani B. Transcriptome of Cultured Lung Fibroblasts in Idiopathic Pulmonary Fibrosis: Meta-Analysis of Publically Available Microarray Datasets Reveals Repression of Inflammation and Immunity Pathways. Int J Mol Sci (2016) 17(12):2091. 10.3390/ijms17122091 PMC5187891 27983601

[26] Allen JT Knight RA Bloor CA Spiteri MA. Enhanced Insulin-Like Growth Factor Binding Protein-Related Protein 2 (Connective Tissue Growth Factor) Expression in Patients With Idiopathic Pulmonary Fibrosis and Pulmonary Sarcoidosis. Am J Respir Cell Mol Biol (1999) 21(6):693–700. 10.1165/ajrcmb.21.6.3719 10572066

[27] Kono M Nakamura Y Suda T Kato M Kaida Y Hashimoto D. Plasma CCN2 (Connective Tissue Growth Factor; CTGF) is a Potential Biomarker in Idiopathic Pulmonary Fibrosis (IPF). Clin Chim Acta (2011) 412(23–24):2211–5. 10.1016/j.cca.2011.08.008 21864521

[28] Pan L-H Yamauchi K Uzuki M Nakanishi T Takigawa M Inoue H. Type II Alveolar Epithelial Cells and Interstitial Fibroblasts Express Connective Tissue Growth Factor in IPF. Eur Respir J (2001) 17(6):1220–7. 10.1183/09031936.01.00074101 11491168

[29] Lasky JA Ortiz LA Tonthat B Hoyle GW Corti M Athas G. Connective Tissue Growth Factor Mrna Expression is Upregulated in Bleomycin-Induced Lung Fibrosis. 1998. Am J Physiol (1998) 275(2):365–71. 10.1152/ajplung.1998.275.2.L365 9700098

[30] Bonniaud P Martin G Margetts PJ Ask K Robertson J Gauldie J. Connective Tissue Growth Factor is Crucial to Inducing a Profibrotic Environment in “Fibrosis-Resistant” Balb/C Mouse Lungs. Am J Respir Cell Mol Biol (2004) 31(5):510–6. 10.1165/rcmb.2004-0158OC 15256388

[31] Ning W Li CJ Kaminski N Feghali-Bostwick CA Alber SM Di YP. Comprehensive Gene Expression Profiles Reveal Pathways Related to the Pathogenesis of Chronic Obstructive Pulmonary Disease. Proc Natl Acad Sci U S A (2004) 101(41):14895–900. 10.1073/pnas.0401168101 PMC522001 15469929

[32] Pi L Fu C Lu Y Zhou J Jorgensen M Shenoy V. Vascular Endothelial Cell-Specific Connective Tissue Growth Factor (CTGF) is Necessary for Development of Chronic Hypoxia-Induced Pulmonary Hypertension. Front Physiol (2018) 9:138. 10.3389/fphys.2018.00138 29535639 PMC5835098

[33] Saggar R Ross DJ Saggar R Zisman DA Gregson A Lynch JP. Pulmonary Hypertension Associated With Lung Transplantation Obliterative Bronchiolitis and Vascular Remodeling of the Allograft. Am J Transplant (2008) 8(9):1921–30. 10.1111/j.1600-6143.2008.02338.x PMC4207285 18671677

[34] Tissot A Danger R Claustre J Magnan A Brouard S. Early Identification of Chronic Lung Allograft Dysfunction: The Need of Biomarkers. Front Immunol (2019) 10:1681. 10.3389/fimmu.2019.01681 31379869 PMC6650588

[35] Todd JL Wang X Sugimoto S Kennedy VE Zhang HL Pavlisko EN. Hyaluronan Contributes to Bronchiolitis Obliterans Syndrome and Stimulates Lung Allograft Rejection Through Activation of Innate Immunity. Am J Respir Crit Care Med (2014) 189(5):556–66. 10.1164/rccm.201308-1481OC PMC3977710 24471427

[36] Shino MY Weigt SS Li N Palchevskiy V Derhovanessian A Saggar R. The Prognostic Importance of CXCR3 Chemokine During Organizing Pneumonia on the Risk of Chronic Lung Allograft Dysfunction After Lung Transplantation. PLoS One (2017) 12(7):e0180281. 10.1371/journal.pone.0180281 28686641 PMC5501470

[37] Shino MY Li N Todd JL Neely ML Kopetskie H Sever ML. Correlation Between BAL CXCR3 Chemokines and Lung Allograft Histopathologies: A Multi-Center Study. Am J Transplant (2021). 10.1111/ajt.16601 Online ahead of print PMC8502500 33840162

[38] Weigt SS Wang X Palchevskiy V Gregson AL Patel N Der Hovanessian A. Gene Expression Profiling of Bronchoalveolar Lavage Cells Preceding a Clinical Diagnosis of Chronic Lung Allograft Dysfunction. PLoS One (2017) 12(1):e0169894. 10.1371/journal.pone.0169894 28103284 PMC5245825

[39] Danger R Royer PJ Reboulleau D Durand E Loy J Tissot A. Blood Gene Expression Predicts Bronchiolitis Obliterans Syndrome. Front Immunol (2018) 8:1841. 10.3389/fimmu.2017.01841 29375549 PMC5768645

[40] Pain M Royer PJ Loy J Girardeau A Tissot A Lacoste P. T Cells Promote Bronchial Epithelial Cell Secretion of Matrix Metalloproteinase-9 Via a C-C Chemokine Receptor Type 2 Pathway: Implications for Chronic Lung Allograft Dysfunction. Am J Transplant (2017) 17(6):1502–14. 10.1111/ajt.14166 27982503

[41] Veraar C Kliman J Benazzo A Oberndorfer F Laggner M Hacker P. Potential Novel Biomarkers for Chronic Lung Allograft Dysfunction and Azithromycin Responsive Allograft Dysfunction. Sci Rep (2021) 11(1):6799. 10.1038/s41598-021-85949-1 33762606 PMC7990920

[42] Igarashi A Okochi H Bradham DM Grotendorst GR. Regulation of Connective Tissue Growth Factor Gene Expression in Human Skin Fibroblasts and During Wound Repair. Mol Biol Cell (1993) 4(6):637–45. 10.1091/mbc.4.6.637 PMC300970 8374172

[43] Frazier K Williams S Kothapallj D Klapper H Gwtendorst GR. Stimulation of Fibroblast Cell Growth, Matrix Production, and Granulation Tissue Fortnation by Connective Tissue Growth Factor. J Invest Dermatol (1996) 107(3):404–11. 10.1111/1523-1747.ep12363389 8751978

[44] Mori T Kawara S Shinozaki M Hayashi N Kakinuma T Igarashi A. Role and Interaction of Connective Tissue Growth Factor With Transforming Growth Factor-in Persistent Fibrosis. In: A mouse fibrosis model. J Cell Physiol (1999) 181(1):153–9. 10.1002/(SICI)1097-4652(199910)181:1<153::AID-JCP16>3.0.CO;2-K 10457363

[45] Sacreas A von der Thüsen JH van den Bosch TPP Weynand B Verbeken EK Debbaut C. The Pleural Mesothelium and Transforming Growth Factor-B 1 Pathways in Restrictive Allograft Syndrome: A Pre-Clinical Investigation. J Heart Lung Transplant (2019) 38(5):570–9. 10.1016/j.healun.2019.02.001 30819647

[46] Vos R Wuyts WA Gheysens O Goffin KE Schaevers V Verleden SE. Pirfenidone in Restrictive Allograft Syndrome After Lung Transplantation: A Case Series. Am J Transplant (2018) 18(12):3045–59. 10.1111/ajt.15019 30019840

[47] Bennett D Lanzarone N Fossi A Perillo F De Vita E Luzzi L. Pirfenidone in Chronic Lung Allograft Dysfunction: A Single Cohort Study. Panminerva Med (2020) 62(3):143–9. 10.23736/S0031-0808.19.03840-0 32192319

