# Supplementary material for: Connective Tissue Growth Factor Is Overexpressed in Explant Lung Tissue and Broncho-Alveolar Lavage in Transplant-Related Pulmonary Fibrosis
Source: Front Immunol. 2021 May 25;12:661761. doi: 10.3389/fimmu.2021.661761 (PMC8187127; doi:10.3389/fimmu.2021.661761)
Supplement: Supplementary file 1 [file DataSheet_1.docx]

Supplementary Material

**Supplementary Methods**

*Broncho-alveolar lavage protocol*

This entails performing broncho-alveolar lavage (BAL) by instillation of 2x50 ml of saline at room temperature, after placing the flexible bronchoscope in a wedge position in a subsegmental branch of a selected bronchopulmonary segment (i.e. the right middle lobe or lingula). Immediately after each 50 ml aliquot instillation without dwell time, gentle aspiration was applied. The two fractions were subsequently pooled and divided for routine analysis (i.e. culture, total cell count and cell differentiation) and a part for research purposes. The BAL aliquot for research purposes was shortly stored at 4°C, and further processed for long-term research storage. This entailed centrifugation at 1500 rounds per minute for 10 minutes, at 4°C. The supernatants was then pipetted in 2ml Eppendorf tubes and subsequently stored at -80°C.

**Supplementary figures**


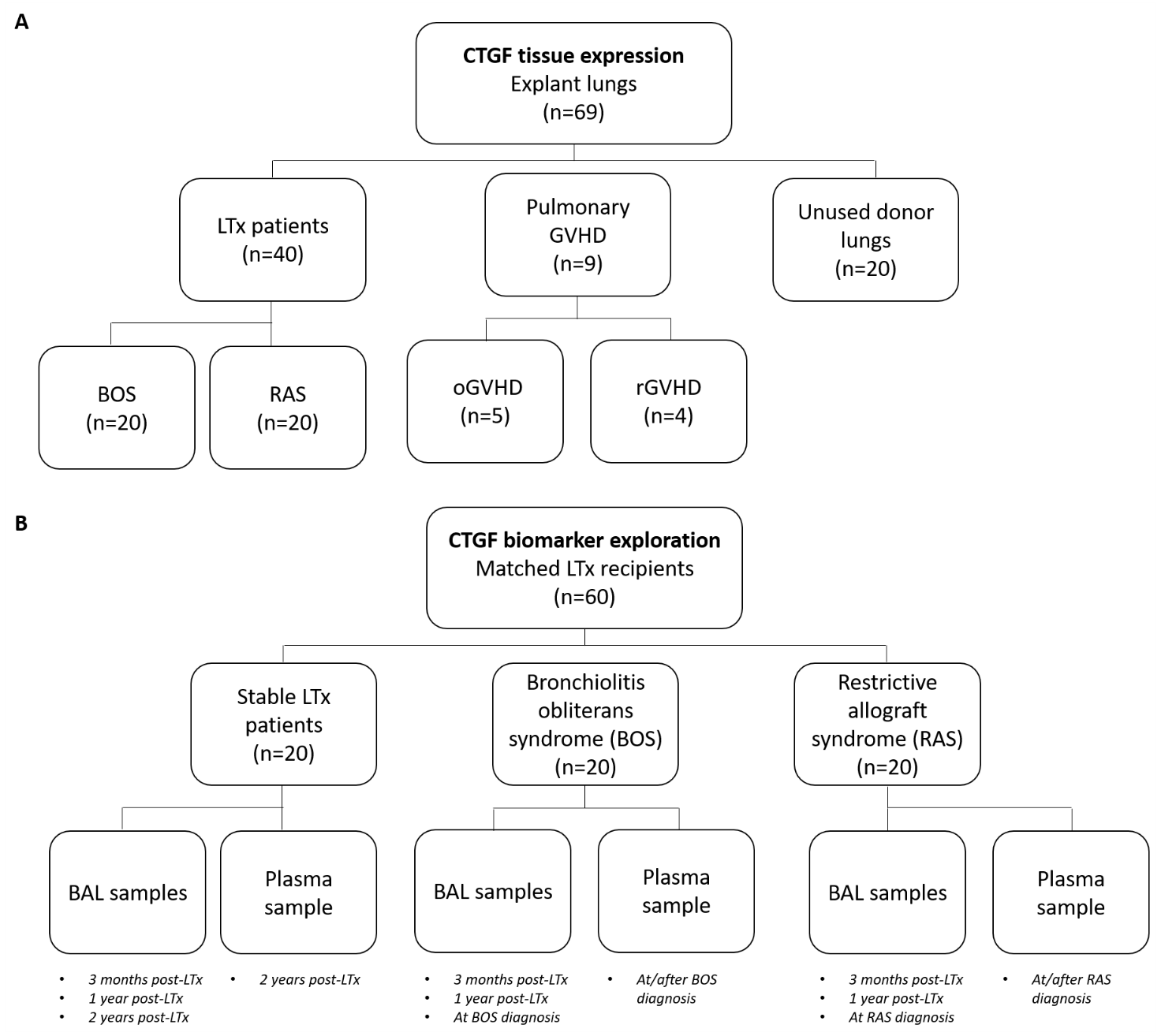


**Supplementary figure S1.** A. Overview of lung tissue collection for qPCR and immunohistochemical CTGF analysis. For 6/20 (30%) BOS lungs and 7/20 (35%) RAS lungs included in the qPCR analysis, no FFPE material was available. B. Overview of BAL and plasma collection from matched BOS, RAS, and stable lung transplant recipients. For 2/20 (10%) BOS patients and 8/20 (40%) RAS patients included for BAL and plasma analysis, explant lung tissue was also available for CTGF qPCR analysis and immunohistochemistry.


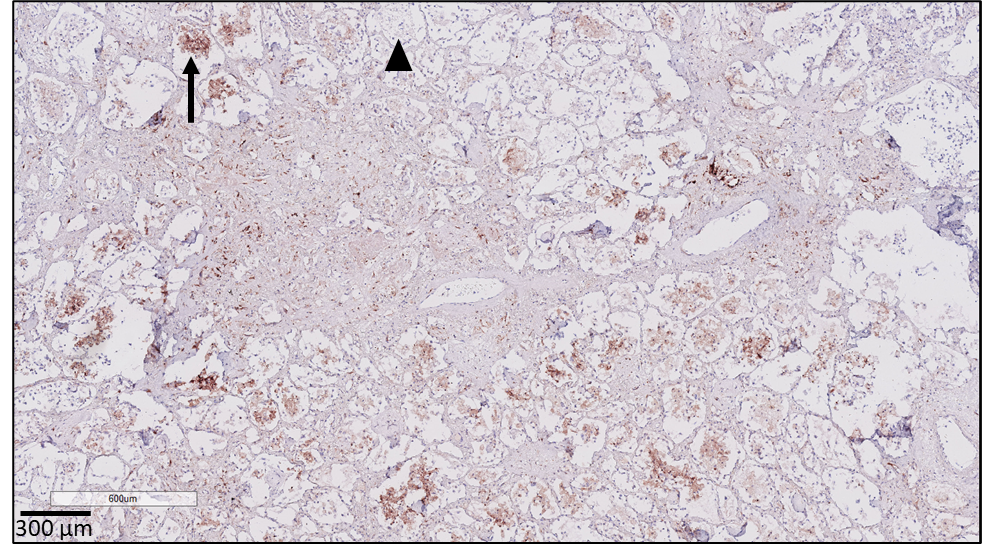


**Supplementary Figure S2.** Representative image of CTGF staining in macrophages of RAS patients, illustrating collections of intra-alveolar macrophages in a zone of interstitial fibrosis with variable staining intensities, ranging from absence of CTGF positivity (arrow head) to strong CTGF positivity (arrow).

**Supplementary Figure S3.** Paired analysis of BAL CTGF values at the different time points with connection of individual paired values. A. Paired CTGF values for stable LTx recipients; there was no difference between different time points (p=0.84). B. Paired CTGF values for BOS patients; no significant difference was observed between different time points (p=0.92). C. CTGF values for RAS patients; CTGF values were significantly higher at CLAD diagnosis (p=0.026); post-hoc analysis confirmed higher CTGF values at CLAD diagnosis compared to 1y post-LTx (p=0.029), but not compared to 3 months post-LTx (p=0.15).

**Supplementary tables**

| **Table S1. Patient characteristics of qPCR analysis** | | | | |
| --- | --- | --- | --- | --- |
|  | **Total** | **Controls** | **BOS** | **RAS** |
| **Patients, N** | 60 | 20 | 20 | 20 |
| **Age at transplant (y)** |  |  | 43 (29-53) | 52 (37-55) |
| **Age at death/graft loss (y)** | 53 (41-61) | 56 (43-66) | 50 (37-59) | 55 (43-60) |
| **Male, N (%)** | 37 (62) | 15 (75) | 9 (45) | 13 (65) |
| **Underlying disease, N (%)** |  |  |  |  |
| Emphysema |  |  | 4 (20) | 8 (40) |
| ILD |  |  | 6 (30) | 6 (30) |
| CF + BRECT |  |  | 8 (40) | 4 (20) |
| PHT + Eisenmenger |  |  | 1 (5) | 2 (10) |
| Other |  |  | 1 (5) | 0 |
| **Type of transplant, N (%)** |  |  |  |  |
| SSLTx |  |  | 19 (95) | 19 (95) |
| HLTx |  |  | 1 (5) | 1 (5) |
| **Type of explant tissue, N (%)** |  |  |  |  |
| Autopsy |  |  | 18 (90) | 13 (65) |
| Redo-transplantation |  |  | 2 (10) | 7 (35) |
| **Time between LTx and CLAD (y)** |  |  | 2.07 (1.12-4.09) | 3.84 (1.54-4.65) |
| **Time between LTx and graft loss (y)** |  |  | 5.53 (3.28-7.72) | 4.66 (3.46-7.20) |
| **Time between CLAD and graft loss** |  |  | 2.53 (1.05-5.10) | 0.97 (0.57-2.40) |

**Supplementary table S1.** Data are shown as n, n (%) or median (interquartile range). CTGF: connective tissue growth factor; BOS: bronchiolitis obliterans syndrome; RAS: restrictive allograft syndrome; ILD: interstitial lung disease; CF: cystic fibrosis; BRECT: bronchiectasis; PHT: pulmonary hypertension; SSLTx: sequential single lung transplantation; HLTx: heart-lung transplantation.

| **Table S2. Patient characteristics of immunohistochemical CTGF analysis** | | | | |
| --- | --- | --- | --- | --- |
|  | **Total** | **Controls** | **BOS** | **RAS** |
| **Patients, N** | 60 | 20 | 20 | 20 |
| **Age at transplant (y)** |  |  | 39 (28-52) | 47 (25-56) |
| **Age at death/graft loss (y)** | 52 (40-60) | 56 (43-66) | 47 (33-57) | 53 (32-60) |
| **Male, N (%)** | 36 (60) | 15 (75) | 9 (45) | 12 (60) |
| **Underlying disease, N (%)** |  |  |  |  |
| Emphysema |  |  | 5 (25) | 8 (40) |
| ILD |  |  | 4 (20) | 6 (30) |
| CF + BRECT |  |  | 9 (45) | 5 (25) |
| PHT + Eisenmenger |  |  | 1 (5) | 1 (5) |
| Other |  |  | 1 (5) | 0 |
| **Type of transplant, N (%)** |  |  |  |  |
| SSLTx |  |  | 19 (95) | 19 (95) |
| HLTx |  |  | 1 (5) | 1 (5) |
| **Type of explant tissue, N (%)** |  |  |  |  |
| Autopsy |  |  | 15 (75) | 13 (65) |
| Redo-transplantation |  |  | 5 (25) | 7 (35) |
| **Time between LTx and CLAD (y)** |  |  | 1.35 (1.01-3.52) | 3.84 (1.03-4.57) |
| **Time between LTx and graft loss (y)** |  |  | 3.96 (2.33-6.72) | 4.62 (2.05-6.02) |
| **Time between CLAD and graft loss** |  |  | 1.90 (0.90-4.64) | 1.06 (0.47-1.94) |

**Table S2.** Data are shown as n, n (%) or median (interquartile range). CTGF: connective tissue growth factor; BOS: bronchiolitis obliterans syndrome; RAS: restrictive allograft syndrome; ILD: interstitial lung disease; CF: cystic fibrosis; BRECT: bronchiectasis; PHT: pulmonary hypertension; SSLTx: sequential single lung transplantation; HLTx: heart-lung transplantation.

| **Table S3. Patient characteristics of patients with pulmonary GVHD after HCT** | |
| --- | --- |
| **Patients, N** | 9 |
| **Age (y)** | 32.85 (20.37 – 44.03) |
| **Gender, male, N(%)** | 6 (67) |
| **Indication for HCT** |  |
| Acute myeloid leukemia | 3 (33) |
| Chronic myeloid leukemia | 3 (33) |
| Myelodysplastic syndrome | 1 (11) |
| Mature T-cell acute lymphoid leukemia | 1 (11) |
| Hodgkin lymphoma, nodular sclerosing type | 1 (11) |
| **Time between HCT and pulmonary GVHD (y)** | 0.84 (0.43 – 2.17) |
| **Time between pulmonary GVHD onset and LTx**  **and lung transplant/autopsy* (y)** | 3.35 (1.45 – 18.10) |
| **Extra-pulmonary GVHD locations** |  |
| Oral | 3 (33) |
| Skin | 2 (22) |
| Gastro-intestinal | 2 (22) |
| Liver | 1 (11) |
| Kidney | 1 (11) |
| Eye | 1 (11) |
| Vagina | 1 (11) |
| Joints | 1 (11) |
| **Pulmonary function test at last follow-up** |  |
| FEV1 (L) | 0.66 (0.50 – 1.08) |
| FEV1 (% predicted) | 21 (14 – 31) |
| FVC (L) | 1.36 (1.14 – 2.57) |
| FEV1/FVC | 0.50 (0.37 – 0.72) |
| TLC (% predicted) | 76.6 (59.65 – 102.00) |

**Table S3.** Patient characteristics of patients with graft-versus-host-disease after allogeneic stem cell transplantation. Data are shown as n, n (%) or median (interquartile range). (*) lung tissue was collected at the moment of lung transplantation for 8 of 9 patients, and at autopsy for 1 of 9 patients. HCT: hematopoietic stem cell transplantation; GVHD: graft-versus-host-disease.

| **Table S4.** **Overview of BAL, plasma, and immunohistochemical CTGF findings in BOS, RAS, stable lung transplant recipients and non-transplanted donor lungs**. | | | | | |
| --- | --- | --- | --- | --- | --- |
| **Explant lung tissue** | **Donor lungs** | **BOS** | **RAS** | **p value** |  |
| **Patients, N** | 20 | 20 | 20 |  |  |
| **CTGF mRNA levels (fold change)** | 1.14 [0.72-1.63] | 2.04 [1.22-3.34] | 1.89 [0.78-3.05] | **0.012** |  |
| **Staining of respiratory epithelium, %** |  |  |  |  |  |
| Positive (≥ 1+) | 30.34 [12.55-37.75] | 77.81 [60.73-90.52] | 95.53 [75.05-98.84] | **<0.0001** |  |
| Mild positive (1+) | 15.25 [9.30-21.85] | 33.46% [20.76-57.62] | 43.09 [20.53-60.38] | **<0.0001** |  |
| Moderate positive (2+) | 6.70 [1.84-8.32] | 14.95 [8.87-17.75] | 20.50 [13.69-23.49] | **<0.0001** |  |
| Strong positive (3+) | 5.85 [1.34-7.82] | 10.90 [5.74-24.24] | 13.43 [8.46-31.60] | **0.0015** |  |
|  |  |  |  |  |  |
| **BAL / plasma** | **Stable LTx** | **BOS** | **RAS** | **p value** |  |
| **Patients, N** | 20 | 20 | 20 |  |  |
| **BAL CTGF expression (ng/ml)** |  |  |  |  |  |
| 3 months post-LTx | 0.11 (0.064-0.17) | 0.13 (0.094-0.19) | 0.18 (0.11-0.37) | **0.025** |  |
| 1 year post-LTx | 0.11 (0.08-0.20) | 0.14 (0.093-0.19) | 0.16 (0.12-0.22) | 0.22 |  |
| CLAD diagnosis / 2 years post-LTx | 0.12 (0.086-0.15) | 0.14 (0.10-0.18) | 0.48 (0.14-0.77) | **0.0009** |  |
| **Plasma CTGF expression (ng/ml)** | 30.60 (26.85-39.25) | 35.30 (25.38-43.45) | 30.45 (22.40-40.70) | 0.74 |  |
| **eGFR (CKD-EPI) (ml/min/1.73 m^2^)** | 54 [38-66] | 54 [33-82] | 55 [38-71] | 0.80 |  |
| **Plasma creatinine (mg/dl)** | 1.34 [1.09-1.62] | 1.16 [0.94-1.86] | 1.32 [0.97-1.69] | 0.95 |  |

**Supplementary table S4.** Data are shown as n, n (%) or median (interquartile range). BAL: broncho-alveolar lavage; CTGF: connective tissue growth factor; BOS: bronchiolitis obliterans syndrome; RAS: restrictive allograft syndrome. eGFR: estimated glomerular filtration rate; CKD-EPI: Chronic Kidney Disease Epidemiology Collaboration formula), qPCR, quantitative real-time polymerase chain reaction.

| **Table S5. Patient characteristics – broncho-alveolar lavage and plasma analysis** | | | | |
| --- | --- | --- | --- | --- |
|  | **Total** | **Stable** | **BOS** | **RAS** |
| **Patients, N** | 60 | 20 | 20 | 20 |
| **Age at transplant (years)** | 55 (48-60) | 56 (45-60) | 53 (40-58) | 55 (49-60) |
| **Male, N (%)** | 26 (43) | 10 (50) | 8 (40) | 8 (40) |
| **Underlying disease, N (%)** |  |  |  |  |
| Emphysema | 37 (62) | 12 (60) | 12 (60) | 13 (65) |
| ILD | 12 (20) | 3 (15) | 3 (15) | 6 (30) |
| CF + BRECT | 5 (8) | 4 (20) | 1 (5) | 0 |
| Redo transplant | 3 (5) | 0 | 3 (15) | 0 |
| PHT + Eisenmenger | 3 (5) | 1 (5) | 1 (5) | 1 (5) |
| **Type of transplant, N (%)** |  |  |  |  |
| SSLTx | 57 (95) | 20 (100) | 17 (85) | 20 (100) |
| HLTx | 1 (2) | 0 | 1 (5) | 0 |
| SLTx | 2 (3) | 0 | 2 (10) | 0 |
| **Time between LTx and CLAD (y)** |  |  | 3.10 (1.46-4.58) | 3.38 (1.81-4.60) |
| **Time between LTx and graft loss (y)** |  |  | 9.12 (5.40-11.86) | 4.59 (2.97-7.34) |
| **Time between CLAD and graft loss** |  |  | 5.32 (2.73-7.03) | 1.19 (1.02-2.70) |

**Table S5.** Patient characteristics of patients included for CTGF protein analysis in broncho-alveolar lavage and plasma. Data are shown as n, n (%) or median (interquartile rangeBOS: bronchiolitis obliterans syndrome; RAS: restrictive allograft syndrome; ILD: interstitial lung disease; CF: cystic fibrosis; BRECT: bronchiectasis; PHT: pulmonary hypertension; SSLTx: sequential single lung transplantation; HLTx: heart-lung transplantation; SLTx: single lung transplantation.
